# Older patients’ experience of primary hypothyroidism: A qualitative study

**DOI:** 10.1111/hex.12656

**Published:** 2018-02-21

**Authors:** Lorna E. Ingoe, Janis Hickey, Simon Pearce, Tim Rapley, Salman Razvi, Scott Wilkes, Susan Hrisos

**Affiliations:** ^1^ Department of Diabetes & Endocrinology Queen Elizabeth Hospital Gateshead UK; ^2^ British Thyroid Foundation Harrogate UK; ^3^ Institute of Genetic Medicine Centre for Life Newcastle University Newcastle upon Tyne UK; ^4^ Endocrine Unit Royal Victoria Infirmary Newcastle upon Tyne UK; ^5^ Institute of Health & Society Newcastle University Newcastle upon Tyne UK; ^6^ Faculty of Applied Sciences Department of Pharmacy, Health and Wellbeing University of Sunderland Sunderland UK; ^7^ Coquet Medical Group Amble Health Centre Amble UK

**Keywords:** experience, hypothyroidism, older adults

## Abstract

**Background:**

Primary hypothyroidism is a common endocrine disorder, more so in an increasing UK ageing population. There is no qualitative research examining the older patient perspective of symptoms, treatment and self‐management of hypothyroidism.

**Objective:**

In this study we explored the experience of hypothyroidism in older people and examined how this may influence their understanding and acceptance of diagnosis, treatment with Levothyroxine and the monitoring process.

**Design:**

We conducted semi‐structured interviews with 18 participants aged between 80 and 93 years. Interview transcripts were analysed using a thematic approach.

**Results:**

The themes involved older individuals’ knowledge about symptoms, confidence in diagnosis and understanding of clinical management regimen to understand hypothyroidism. Interpretation of the themes was informed by the Health Belief Model.

**Conclusion:**

Our findings can help to inform the development of interventions by treating clinicians and support staff to engage older patients in the long‐term management of this chronic condition.

## 1 INTRODUCTION

Primary hypothyroidism is a common disorder affecting all age groups with a higher frequency in women and older individuals. In the UK, the Whickham Study found that 10% of the population above the age of 75 years had a raised thyroid stimulating hormone (TSH) level.^1^ Currently most individuals with hypothyroidism are treated with Levothyroxine sodium (LT4) as replacement therapy. The starting dose of LT4 varies and is altered in 25‐50 mcg increments at two to three monthly intervals^2^ to achieve a target serum TSH level within the local reference range. The correct dose is that which enables the patient to achieve a euthyroid state and symptomatic relief.^3^ Further LT4 dose adjustment may be required due to changes in body weight, advancing age,^4^ concomitant medication or co‐morbidities. Ongoing review should include an estimation of serum TSH level annually^2^ and a clinical assessment of wellbeing.^5^

The dearth of published research relating to hypothyroidism in older patients significantly limits understanding and management of the condition in this age group. Hypothyroid individuals havereported moretiredness, weight gain, brittle nails, dry skin and hair, than euthyroid individuals.^6^, ^7^ It is acknowledged that these classic symptoms of hypothyroidismcan be vague and ambiguous.^8^ This association has not been specifically observed in older people. Existing literature also focuses on clinical aspects of hypothyroidism such as its negative impact on cognitive function,^9^ adverse psychological wellbeing^10^ or long term complications of over‐treatment with LT4 such as bone fracture.^11^ It has been recommended that in patients over 85 years of age the initial replacement LT4 dose should be lower and there should be smaller increments in dose adjustment to avoid cardiac complications.^12^

Two interview studies, again focusing on younger individuals, looked at patient perspectives of hypothyroidism. In a convenience sample of twelve females aged between 32 and 68 in Canada patients described initial and ongoing symptoms despite treatment with thyroid hormone replacement and the experience of diagnosis. Patients continued to seek validation of their experience at diagnosis and during the treatment phase.^8^ Nexo et al^7^ explored the experiences of five people aged between 18 and 65 years with auto‐immune hypothyroidism in Denmark with results echoing those of the earlier study. It is not known how relevant this information is to the experiences of older individuals. This is important because preliminary evidence is emerging to suggest that current guidelines for the management of hypothyroidism may not be appropriate for older patients. Different TSH reference ranges may be required for elderly patients to prevent excess mortality.^13^, ^14^

We carried out an interview study alongside a feasibility randomised controlled trial (RCT) to examine the impact of reducing the dose of LT4 in patients aged 80 and over with an existing diagnosis of primary hypothyroidism.^15^ Whilst the purpose of this embedded study was to explore barriers and facilitators to recruitment and retention of older patients in a clinical trial, interviewees’ narratives incidentally provided a unique insight into their experiences and beliefs in relation to hypothyroidism. This paper reports these novel findings and discusses thisdata relating to older individuals with a chronic condition within the framework of the Health Belief Model (HBM).^16^

## 2 METHODS

### 2.1 Ethics

Favourable opinion was obtained from Sunderland NHS research ethics committee on 03/04/2012 (reference 12/NE/0098) for this study that took place in the North East of England. All participants provided written informed consent.

### 2.2 Design

This is a qualitative study using a semi‐structured topic guide. Interviews were undertaken by LI and took place between November 2012 and December 2013. Interviews were audio recorded and transcribed verbatim. All patient/personal identifiers were removed or disguised in the transcripts so the patients described are not identifiable and cannot be identified through the details of the story.

### 2.3 Participants

Participants were eligible if they had a diagnosis of primary hypothyroidism for at least 6 months treated with LT4, TSH levels within the local reference range and were community dwelling. Exclusion criteria were: previous diagnosis of thyroid cancer, inability to provide informed consent, or risks to the researchers visiting the participant at their home.

### 2.4 Procedure

Participants were approached after agreeing to randomisation on the SORTED 1a RCT (“Accepters”) or from a group of patients who had declined the trial (“Decliners”). Accepters were purposively sampled for the interview study based on characteristics such as recruiting centre, location of study visits (home or hospital), geographical spread (rural or urban) and gender. Purposive sampling was planned for Decliners but recruitment proved difficult so these patients were approached sequentially. All approached trial Accepters agreed to take part in an interview (n = 11) and of the 24 Decliners approached, seven agreed to be interviewed. We interviewed participants individually or with a significant other present such as spouse or adult offspring. We did not consider conducting focus groups as the majority of participants had requested domiciliary visits for the RCT. One interview was conducted in a hospital Out‐Patient department at the participants’ request and the others were held at participants’ homes. Interviews were completed within 4 weeks of accepting or declining trial participation to help with recall and Accepters had a further interview once RCT participation had ended in order to reflect upon trial involvement. Interviews lasted an average of 35 minutes. They were deliberately kept short and had a conversational style to maintain the patient's focus and prevent fatigue. A topic guide was followed with open ended, neutral and sensitive questions that primarily explored patient's reasons for agreeing and declining to participate in the RCT. Respondents were also asked about diagnosis, symptoms, elements of self‐management and treatment with LT4. Questions were informed by a review of relevant literature. Patient narratives in response to these questions were typically embedded within their experience of living with hypothyroidism. Areas of discussion brought up by the participants were then examined in greater depth.

### 2.5 Data analysis

Initially LI became immersed in the data by conducting and transcribing the interviews and reading through the manuscripts and reflexive notes. Emergent themes were discussed in depth with SH and TR and agreement was high. We developed a coding frame to organise the data into meaningful groups. Themes were identified as the aspects of data that captured something new or important in relation to the older person's experience of hypothyroidism. Formal analysis used open and focused coding, constant comparison, deviant case analysis and memoing.^17^ Codes were added or revised as new themes and sub‐themes became apparent. The transcripts were annotated with these codes. Interviews continued until data saturation was reached and no new themes were emerging.^18^ All transcripts were reviewed by SH. Preliminary findings were discussed during regular team meetings that included a patient and public involvement member (JH), and clinical representatives working in primary and secondary care managing patients with hypothyroidism (SR, SP & SW). A thematic chart using an EXCEL package provided an overview of the entire data set. The chart was organised into three themes. The credibility of the analysis was agreed upon by LI, SH and TR. Participant quotes were used to illustrate the themes.

Two models were identified that had potential to aid interpretation and application of our interview findings. To help explain health behaviour for older patients with hypothyroidism emergent themes from the interview data were mapped to dimensions of the HBM. The HBM states that health behaviour is determined by personal beliefs or perceptions about a disease and the strategies used to reduce its occurrence. It is well established and evaluated, and is constructed around four main elements: Perceived Seriousness; Perceived Susceptibility; Perceived Benefits and Perceived Barriers. The Healthy Ageing Model^19^ focuses on active engagement in health care for older adults with chronic health conditions managed in primary care, and provides a framework to guide healthcare professionals in delivering supported self‐management.

## 3 RESULTS

The characteristics of the Accepters and Decliners are shown in Tables 1 and 2 respectively. There were thirteen female and five male participants. Median age was 83 years (range 80‐93 years) and median length of diagnosis of hypothyroidism was 14 years (range 3‐24 years). Both groups reported similar experiences of diagnosis, treatment and management of hypothyroidism. Three key themes emerged: a general lack of knowledge about the symptoms of hypothyroidism and the circumstances surrounding diagnosis which were sometimes vague; treatment was understood in terms of biochemical management such as the reliance on annual blood tests, the tablets required for its control and past experience of dose changes; ongoing management was generally conveyed in terms of trust in the General Practitioner’s (GP) management of the condition.

**Table 1:** Key participant characteristics (Accepters)

Gender	Age (years)	Length of diagnosis of hypothyroidism (years)
Female	93	13
Female	80	10
Male	84	Approx. 8
Female	82	24
Male	83	15
Female	88	9
Female	85	14
Female	80	3
Female	83	14
Female	82	18
Female	85	16

**Table 2:** Key participant characteristics (Decliners)

Gender	Age (years)	Length of diagnosis of hypothyroidism (years)
Male	85	16
Female	Early 80s	Approx. 5
Male	82	29
Male	85	5‐10
Female	Early 80s	Approx. 20
Male	80	More than 10
Female	83	14

### 3.1 Understanding hypothyroidism in terms of symptoms

Six participants had presented with manifest symptoms to their GP before diagnosis. Some had presented with tiredness which became gradually noticeable and necessitated multiple appointments until a diagnosis was made. Two female participants recounted how they had experienced more profound warning signs that had impacted significantly on their quality of life. These ladies, who had been diagnosed many years ago when in their fifties, gave very similar accounts surrounding the presentation of their symptoms. These participants reported that their GP attributed symptoms to the ageing process and in particular to menopause. Consequently both participants had delayed diagnoses which had caused them distress. The effect of thyroid replacement treatment on these participants’ symptoms was reported to be immediate and dramatic. Some of the remainder reported being unaware of any obvious symptoms, describing their diagnosis as an incidental finding following a routine blood test. It is of course feasible that GPs recognised or elicited experience of symptoms in their discourse with patients, and then instigated appropriate investigations to exclude thyroid disease without relaying this information to the patient.

“…One of my problems before I was diagnosed was falling asleep all over. Falling asleep in the middle of a dinner party, falling asleep at the dinner table falling asleep driving the car and yeah I was working and it would affect my decisions that I made”. *Female aged 82*

“…I started to feel… that I was losing it altogether. I couldn't speak properly and my features changed and my hair and my eyebrows and I felt cold the whole time and apparently so my family tell me I was very bad tempered, got into rages and I used to sit in my dressing gown and didn't do anything just by the fire tried to keep warm…the doctor… decided I was suffering from an early menopause… but it just went on and on and on…” *Female aged 85*

“…I remember it was like being reborn. It was a new life. It was just the most the best thing that had happened to me. It was amazing….” *Female aged 82*

“…I went to the doctors for something I can't remember what it was and he said I think it's time you had an MOT. And then they took blood tests and that's how they discovered I had problems with my thyroid”. *Female, aged 80*

Some participants appeared to be uncertain about the relationship of their symptoms to hypothyroidism and persisting symptoms were not perceived as problematic (in terms of their hypothyroidism) once treatment with LT4 had commenced. These beliefs were often reinforced by similar reflections made by family members or friends.

“Well she [friend] says your hair's not very thick and I says I know but that's probably old age. You know it gets thinner as you get older”. *Female aged 80*

“My Mam feels the cold…it might not be the thyroid…it could be just your circulation”. *Daughter of Female aged 82*

### 3.2 Understanding hypothyroidism in terms of medication

Several participants describe similar titration of LT4 doses after diagnosis with some followed by occasional dose adjustments. Once established on LT4 treatment adjustments were in the form of a reduction in dose for a very short period of time then an increase again to the previous level. Sometimes a dose increase (25 mcgs daily) or a prescription for a slight overall increase in the form of a variable daily dose may occur.

When GPs had increased the dose of LT4 during long term treatment there was a tendency among participants to view this as an advantageous event. Often participants had been explicitly told that an increase would have direct health benefits. One participant who had significant signs and symptoms at diagnosis (Female aged 85) made an implicit assumption that higher LT4 doses are associated with a better quality of life. Another had had a recent slight reduction in LT4 dose but concluded this may be a temporary measure.

“Well I was happy in a sense that [GP] said I would benefit from it. And that I would perhaps lose a bit of weight because of going onto it. And I believe that's true. I've lost maybe a couple of stone”. *Male aged 80*

“…I feel that I'm probably more with it than I would be if…perhaps the correct dosage of thyroxine perhaps makes me a little better than I would be if I was just a normal person with a normal thyroid. It's keeping me topped up if you like perhaps…beyond my age…” *Female aged 85*

“…Cos the thing I would have thought that with ageing you might need more [LT4] …” *Female aged 82*

“…I was taking 75 and I went to the doctors and he said…with all your joint pains and everything you're getting…sometimes the thyroid tablet…can cause joint pains if you're taking too much or too little… he did the blood tests again and then he rang me up and he said…we're going to reduce it to 50… *Female aged 80*

Some participants believed that LT4 is an essential and beneficial medication although the “silent” nature of hypothyroidism had caused others to doubt the veracity of the diagnosis or to question the need to continue to take thyroid replacement treatment. The number and type of prescribed medicines can create other everyday anxieties. For example participants worry that they may take the wrong dose or the wrong tablet. Most took LT4 in a fixed pattern once daily and generally organised around the daily routine of getting up so that they remembered to take it.

“If I have an under active thyroid which makes you feel cold I'd like the best possible treatment and doses. And the present doses don't make me any warmer if I can put it like that. So therefore I wouldn't really want to reduce the dose on that basis”. *Male aged 85*

“I don't think the thyroid tablets actually make a difference. I have no ill effects from the thyroid.” *Female aged 80*

“Because I was taking 75 and there aren't 75 milligram [microgram] pills you have to take a 25 and a 50… and as they're the same pill in the same colour and the same size for somebody of my age with failing sight… it's not easy… I've got to be sure that I take one out of that packet and one out of that packet. Not two out of that packet because then I could be taking a 100 or I could be taking only 50. I don't know”. *Male aged 83*“… I take it about seven o'clock half past seven in the morning. I have it by my bed… otherwise you might forget it…” *Female aged 93*

### 3.3 Understanding hypothyroidism in terms of ongoing management

Apart from taking daily medication the main functional effect on the participant of an under‐active thyroid appears to be its management. Participants describe ongoing management as appointments once or twice yearly to have a blood sample taken. The purpose of the blood sample was commonly perceived as a check on the thyroid. One participant mentioned blood thyroid levels but none recollected a direct symptom check in association with reviews and medication adjustments. Participants did not generally associate symptom checks with ongoing management although it remains possible that the GP took this into consideration when making treatment changes without necessarily imparting this to the patient.

“I haven't got a problem really. I wouldn't know I had unless it was the blood tests that's all”. *Female, early 80s*

“it's just a yearly check I mean I don't think that's often enough….I wouldn't like to feel that I'd gone for a year and then found…I should've been on a different dosage…for the thyroid problems.” *Female aged 85*

“they check it every time they take some blood. They check the thyroid everything's checked…” *Female aged 83*

“…It was because of a blood test I'd had and the blood test maybe for several different reasons. And the thyroid was obviously one of them. And he [GP] decided that I should go on to the higher dose”. *Male aged 80*

Participants appeared willing to engage in the monitoring programme because they had confidence in its effectiveness but were sometimes worried about the results of blood tests. For example, participants recounted interactions with health care workers where feedback garnered about results was limited sometimes leading to feelings of anxiety or frustration. They describe a tendency to receive limited explanations of the reasons for altering LT4 dosage and little or no autonomy in decision‐making and control over LT4 dosage. Lack of information sharing, including being unsure about why the dose of LT4 would be altered, seems to either cast doubt on the diagnosis of hypothyroidism or the necessity of taking medication or lead to passive acceptance. Consequently some participants seek out information from informal sources to fully understand diagnostic decisions and treatment changes.

“… I don't understand why it [LT4 dose] would or wouldn't be…[altered]” *Female, early 80s*

“Oh I think [its] at least three year since it's been adjusted. But I'm always told about it…there's no details except [that] it would be better – “we are going to increase your dosage. But don't worry”. *Female aged 85*

“You know we weren't aware that he had any thyroid problems until the surgery did a blood test and I don't think anything's been said about it from the surgery ever…well they just prescribed the thyroid things [LT4]. So we just thought that because we didn't know so much it would be a good idea to do this [participate in RCT]. We could find out at the same time whether he actually needs to take it [LT4]…” *Wife of male aged 84*

“I don't really know what the symptoms were I wasn't told what the symptoms were…I mean it wasn't explained by the doctor nor nothing.. I didn't even know what my thyroid was …..I've just kind of accepted it it's just been since then part of my life.” *Female aged 80*

Participants acknowledged trust in their GP and expressed a belief that the GP's prime consideration was for their general welfare despite feeling vulnerable in the clinical process. Underlying the ongoing monitoring process is a sense of stoicism and to a certain extent a passive acceptance of the status quo.

“He [GP] didn't say anything he just said your thyroid's struggling go on to 75…I just do what the doctor says*”. Male aged 82*

“…If it was explained to you…when they were doing the things you would understand it a little bit more…You see I'm not stupid and I like to know the reason why”. *Female aged 80*

“…I was on 100 a day and then he put it up put me up to the 200 on a Monday and that seems to have stabilised me. Hopefully…he's [GP] just sort of got me stabilised…” *Female aged early 80s*

“…I'm a great believer in they're trying to help you. There's only one way you've got to do what they say. And I'm a great believer in that”. *Female aged 82*

## 4 CONSIDERATION OF EMERGENT THEMES WITHIN THE FRAMEWORK OF THE HEALTH BELIEF MODEL

Knowing how older patients understand their hypothyroidism has important implications for how this condition is managed within this age group and will be particularly important if we need to evaluate an age related TSH reference range in the future.^14^ Our findings suggest significant gaps in older participants’ knowledge and understanding of the condition, its symptoms and its management. To better understand the implications of this the emergent themes were considered within the framework of the HBM.

According to HBM, how a person views their susceptibility or personal risk to a particular disease or condition is one of the most powerful perceptions in prompting them to adopt healthier behaviours. Participants in the current study rarely discussed their hypothyroidism in relation to symptoms but instead tended to talk in terms of genetic predisposition or of an inevitable effect of ageing. Consequently the impact of ongoing symptoms such as tiredness, sensitivity to the cold and those related to appearance such as thinning hair and brittle nails tended to be minimised or to be attributed to other causes such as advancing age. Similarly, symptoms of weight gain were attributed to being less active and/or to other diagnosed co‐morbidities. Their perceived susceptibility of the condition was therefore firmly embedded in being in a euthyroid state, determined by a blood test, regardless of the presence of manifest symptoms.

A further core HBM construct is perceived seriousness, which is the individual's judgement as to the severity of their condition. Our findings suggest that for the older participants we interviewed, their perceived severity of hypothyroidism is influenced by their initial experience of receiving a diagnosis, in particular whether or not their diagnosis came about following significant manifest symptoms or was an incidental finding following what appeared, to them at least, a routine blood test. The circumstances surrounding diagnosis also influenced participants’ beliefs about the presence of disease, with some questioning the need for medication and others demonstrating an expectation that a reduction in LT4 dose would ultimately result in a return of symptoms.

**Figure 1:**
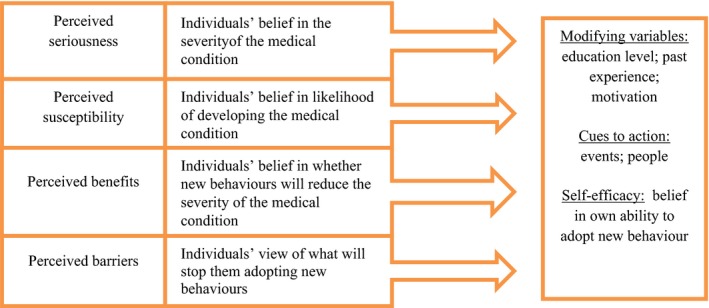
The Health belief model

Conceptualising our findings in this way already begins to illuminate potential perceived benefits and perceived barriers, the remaining two core constructs of the HBM, which are likely to influence the appropriate management, or changes to the management of hypothyroidism in older patients. The model is suggesting for example, that patients who perceive benefits of being on a higher dose of LT4 for the suppression of symptoms may perceive a reduction in LT4 dose as a ‘threat’, which will in turn influence the likelihood of them being receptive to a reduction in dose (Figure 1). Similarly, perceived barriers to ongoing management appear to be a lack of understanding of hypothyroidism and the significance of TSH levels and their relationship to LT4 doses. Importantly, the beliefs captured within these constructs are, theoretically, amenable to change and the framework provides a basis for informing the development of interventions to support behaviour change and improved management of hypothyroidism in this age group.

In addition to these four core constructs, the HBM further proposes a number of modifying factors (personal factors relating to the individual), cues to action (factors that prompt the person to do something), and self‐efficacy (the individual's belief in their ability to do something) that can influence the individual's perceptions of susceptibility and illness severity. HBM helps to illustrate theoretically how patients’ disconnection between on‐going symptoms and the stability of their hypothyroidism may inhibit them acting on manifest symptoms as ‘cues to action’ to improve the management of ongoing symptoms. The patients also seemed to lack self‐efficacy, tend not to challenge medical practitioners and accept prioritisation of TSH control over symptom relief.

## 5 DISCUSSION

For this sample of participants, perceptions surrounding diagnosis, treatment and management of hypothyroidism tended to lead towards a belief that this disease is relatively symptom free, has a simple remedy and a predictable management regime which does not require behaviour modification. No one expressed any ill effects from taking LT4 and the therapy appears to be convenient and achieves the desired therapeutic effect. Treated hypothyroidism causes no major disruption to patients’ lives, there are no flare‐ups and the managed disorder appears to be fairly predictable and certain.

There is an implicit acknowledgement that management of an under‐active thyroid is biochemical rather than symptomatic. Symptoms were described as being few in number and were commonly attributed to other causes, demonstrating participants’ uncertainty about the relationship of known common or classic symptoms to hypothyroid diagnosis. This assumption is perpetuated by the resignation that their symptoms cannot be managed by clinicians because the blood results are within normal parameters. Symptoms are largely self‐managed and participants have accepted that complete relief is not always possible.

Although GPs may be discussing symptoms with patients during appointments, patients may not recognise this. An acknowledgement of the existence of possible symptoms and some advice or treatment may lessen their impact. The relationship between symptoms, hypothyroidism and activities of daily living and hence physical and emotional functioning in this age group are not well understood. Our findings suggest that a combination of circumstances can lead to a lack of anxiety about hypothyroidism and other established long‐term conditions take precedence especially if they limit functionality.

By understanding prescribed medication and its use, older people could assume responsibility and contribute to decisions in relation to their health care. An important aspect of enabling and encouraging self‐care for older people may be to provide education catered to the individual. A key aspect of the Healthy Ageing Model^19^ is the health professional acting as a coach rather than solely as a source of information. The clinician helps to set individualised, achievable goals in conjunction with the patient in the primary care setting. Our findings suggest that patients in this age group do want to understand hypothyroidism and its management, especially when LT4 doses are adjusted. However, they do not understand dose changes. An explanation of the relationship between biochemical markers and dosage could address this uncertainty and aid patients’ understanding. Patients’ past experience and sense of coherence shows that as they age they tend to take more medication for other co‐morbidities and the dosage of medication also increases. Although participants state that they are not concerned about a change in LT4 dose on their health status, they assume that they would feel worse if the dose was reduced. If LT4 doses always increase then this may have subtle effects on patients’ understanding of the disease. The participants who have had LT4 dose reductions in the past have felt some anxiety and assumed that it would be a temporary measure. These findings all have implications for the successful and appropriate management of older patients with hypothyroidism.

However in hypothyroidism patients generally assume that their biochemical results do not vary and annual monitoring is sufficient. When biochemical results are reported as being normal this creates an uncertainty with regard to ongoing symptoms. There is a disconnection on the part of the patient with regard to ongoing management of this condition where they strongly report the perspective of the clinician. This results in little inclination to disengage from the consultative process. Participants trust their GP to prescribe the LT4 dosage that they require.

Mapping our findings to the HBM allowed us to structure the preliminary understanding gained from our participant interviews within a robust theoretical framework of health related behaviour. This analysis provides the basis for generating hypotheses and will be used to guide further exploratory work in this area. Assessing older patients’ personal models of other chronic conditions (diabetes) has been seen to be valuable in promoting self‐management especially through the identification of beliefs regarding the effectiveness of treatment which were predictive of dietary intake and physical activity. Understanding illness models can create individualised education and counselling sessions.^20^

The participants in this study described being passive in the medical decision making process in relation to their hypothyroidism and tended to give it a low priority amongst other health status variables. The assignment of low priority, perhaps because of the lack of information surrounding diagnosis and feedback regarding results has created a low desire to engage in attempting to understand the disorder in more depth as patients feel that their hypothyroidism seems to be well controlled with an annual blood test and LT4 treatment.

### 5.1 Limitations

This article presents very preliminary data as the study was not designed to examine patients’ experience of living with hypothyroidism so some aspects may not have been elicited. Data saturation was based on the objectives for the RCT so some issues may have been missed. Patients whose recent TSH levels were within the local reference range were invited to participate in the RCT so it is possible that the experience of those with variable TSH levels may have been different.

## 6 CONCLUSION

Though tentative this study provides a unique insight and first report of patient experience in older patients living with hypothyroidism. Our findings reveal a general lack of knowledge of the signs and symptoms of hypothyroidism and reasons for LT4 dose adjustment amongst this group. Older patients may not vocalise or may underplay any symptoms they experience when attending for review. Patients accept the medical decision to adjust LT4 doses and do not ask for explanations even though dose adjustments may not appear to chime with their understanding of hypothyroidism or the ageing process. This may affect compliance with LT4 medication and any future implementation of an age‐related TSH reference range. In seeking to understand dose changes patients search for advice mainly from family members or friends. The analysis of these findings against the HBM has provided guidance for future work with this age group. It is important that patients feel empowered to seek information from treating clinicians. Further research is also required to investigate the attitudes of clinicians towards patient education and their management of hypothyroidism in older patients. It would be useful to discover whether lack of knowledge about hypothyroidism is confined to this age group alone. A cross sectional study of different age groups to ascertain perceptions of hypothyroidism would be useful in understanding the type of patient education required.

## CONFLICTS OF INTERESTS

LI, SH and TR do not have any conflicts of interests. SW & JH hold a research grant with Amdipharm Mercury (AMCo), manufacturers of levothyroxine & JH is Director of the British Thyroid Foundation which has received sponsorship from AMCo for newsletters, and patient education films in 2014. SR receives fees from AMCo for consultancy services. SP has received speaker fees from Merck and ViroPharma.
